# Dynamic changes of amplitude of low-frequency in systemic lupus erythematosus patients with cognitive impairment

**DOI:** 10.3389/fnins.2022.929383

**Published:** 2022-08-23

**Authors:** Yifan Yang, Ruotong Zhao, Fengrui Zhang, Ru Bai, Shu Li, Ruomei Cui, Shuang Liu, Jian Xu

**Affiliations:** ^1^Department of Rheumatology and Immunology, First Affiliated Hospital of Kunming Medical University, Kunming, China; ^2^Department of Magnetic Resonance Imaging, First Affiliated Hospital of Kunming Medical University, Kunming, China

**Keywords:** systemic lupus erythematosus, cognitive impairment, resting-state fMRI, dynamic amplitude of low-frequency fluctuation, static amplitude of low-frequency fluctuation

## Abstract

**Background:**

Cognitive dysfunction (CI) is frequently reported in patients with systemic lupus erythematosus (SLE), but the identification and assessment of SLE-related CI remain challenging. Previous studies have focused on changes in static brain activity, and no studies have investigated the characteristics of dynamic brain activity in SLE patients with CI.

**Objects:**

We calculated the dynamic amplitude of low-frequency fluctuation (dALFF) by combining the ALFF with a sliding window method to assess the temporal variability of brain functional activity in SLE patients with and without CI.

**Methods:**

Thirty-eight SLE with CI, thirty-eight SLE without CI, and thirty-eight healthy controls (HCs) were recruited. By comparing static ALFF (sALFF) and dALFF among the three groups, changes in brain activity intensity and its temporal variability were assessed in patients with SLE with or without CI. Spearman correlation coefficients were calculated between the brain function indicator and Mini-mental State Examination (MMSE) scores of SLE with CI.

**Results:**

Subjects among the three groups exhibited significant sALFF differences in the right parahippocampal gyrus, left caudate nucleus, right putamen, and left cuneus. Compared to the SLE without CI, the right parahippocampal gyrus exhibited higher sALFF in the SLE with CI group. Compared to the HCs, the left caudate nucleus exhibited increased sALFF in the SLE with CI group. Participants in the three groups exhibited significant dALFF variability in the right parahippocampal gyrus, right lingual gyrus, and bilateral inferior occipital gyrus. Compared to the HCs, the right lingual gyrus exhibited reduced dALFF in the SLE without CI group. Compared to the HCs, the right parahippocampal gyrus exhibited increased dALFF, left calcarine fissure, and the surrounding cortex exhibited reduced dALFF in the SLE with CI group. There was no significant correlation between the MMSE score, sALFF, and dALFF in the SLE with CI group.

**Conclusion:**

SLE patients with CI have abnormal brain activity intensity and stability. By analyzing the dynamics of intrinsic brain activity, it provides a new idea for evaluating SLE-related CI. However, more research and validation with multiple metrics are needed to determine the link between the severity of cognitive impairment (CI) and brain activity in patients with SLE.

## Introduction

Systemic lupus erythematosus (SLE) is a typical autoimmune disease with a global prevalence rate of 0–241/100,000 (Rees et al., 2017). When SLE involves the central and/or peripheral nervous system, it is called neuropsychiatric systemic lupus erythematosus (NPSLE). The clinical manifestations of NPSLE are complex, ranging from mild headache, cognitive impairment (CI), mood disturbance, a series of neurological symptoms, and mental disorders ranging from subtle abnormalities, such as neuritis, to severe manifestations such as epilepsy, cerebrovascular accident, and myelopathy are considered to be the most serious complications and poor prognostic factors of SLE. NPSLE is the second leading cause of death after lupus nephritis in patients with SLE (Schwartz et al., 2019), and the mortality rate is 10 times higher than that of the general population (Zirkzee et al., 2014), and it severely damaged the patients’ quality of life (Ogunsanya et al., 2018). The prevalence of CI in patients with SLE reported in previous studies was highly heterogeneous, ranging from 6.6 to 80.0% (Schwartz et al., 2019), significantly higher than in healthy individuals (Kozora et al., 1996; Al-Homood et al., 2017; Zhang et al., 2017; Shaban and Leira, 2019; Kim et al., 2021). Identifying and assessing SLE-related CI remains challenging at present due to the lack of sensitive and standardized neuropsychiatric tests and diagnostic biomarkers (Seet et al., 2021).

Functional magnetic resonance (fMRI) is divided into resting-state fMRI (rs-fMRI) and task-state fMRI. The most commonly used method is blood oxygenation level-dependent (BOLD) imaging. When the brain neurons are excited, their oxygen consumption increases and the local blood flow in the brain area increases. This process will lead to changes in the ratio of local oxyhemoglobin and deoxyhemoglobin. Oxyhemoglobin is diamagnetic, while deoxyhemoglobin is paramagnetic. BOLD-fMRI uses hemoglobin as an endogenous contrast agent to measure the BOLD signal generated by the difference in the magnetization vector between the two hemoglobins to observe the activity of the brain indirectly, non-invasively, and non-radioactively (Fox and Raichle, 2007; Liu et al., 2018). The commonly used indicators of fMRI include regional homogeneity (ReHo), low-frequency amplitude (amplitude of low-frequency fluctuation, ALFF), fractional low-frequency amplitude (fractional amplitude of low-frequency fluctuation, fALFF), degree centrality (degree centrality, DC), etc. Local brain activity reflects the intrinsic properties of brain tissue activity and is related to psychological and cognitive processes (Britz et al., 2011; Hutchison and Morton, 2016). ReHo is the similarity of the time series of a given voxel to the time series of its nearest neighbors, i.e., the consistency of the functional activity between the local voxel and the adjacent voxels (Zang et al., 2004); ALFF is an indicator that can reflect the characteristics of spontaneous activity of local neurons in the resting state by calculating the power spectrum of low-frequency fluctuation signals with a frequency of 0.08–0.10 Hz (Zang et al., 2007), and fALFF is the ratio of ALFF to the root mean square of full-spectrum power, which can reduce the sensitivity of ALFF to physiological noise (Zou et al., 2008). fMRI has been used to study brain function in diseases including SLE. For example, a systematic review of fMRI studies in patients with SLE found that 72.7% of the literature reported increased brain activity in SLE, including pediatric patients without neuropsychiatric symptoms and patients with disease duration of less than 2 years (Mikdashi, 2016). A resting-state fMRI study of non-NPSLE patients found that ReHo values in the fusiform gyrus and thalamus were decreased, and ReHo values in the parahippocampal gyrus and uncinate gyrus were increased in patients with SLE. The ReHo value of the cerebellum was positively correlated with disease activity and the ReHo value of the frontal gyrus was negatively correlated with disease activity, and some brain regions showed correlation with depression and anxiety states (Liu et al., 2018). Another similar study also found that non-NPSLE patients had abnormal increases or decreases in ALFF, fALFF, and ReHo in multiple brain regions compared with healthy controls (HCs), and these abnormalities were correlated with self-rating anxiety scales (Piao et al., 2021). A task-state fMRI study of non-NPSLE patients found abnormally reduced activation of the limbic system but higher activation of memory, emotion, and behavioral systems in patients with SLE, suggesting that these patients with SLE have subclinical cognitive dysfunction and decision-making deficits (Wu et al., 2018).

Previously, common resting-state fMRI studies defaulted to a constant intensity of brain activity throughout the MRI scan, but more and more studies have shown that even during MRI scans, changes in brain activity over time are dynamic. Brain dynamics-based studies can deepen connections to human brain mechanisms and disease-induced brain damage (Allen et al., 1991; Leonardi et al., 2013; Li et al., 2019). Therefore, some studies have proposed using ALFF combined with dynamic ALFF (dALFF) analysis to study the dynamic changes of local brain activity in the human brain, that is, to study the temporal variation of local brain activity amplitude between voxels by calculating the ALFF changes over time, which helps to improve the reliability of research results (Tagliazucchi et al., 2014; Fu et al., 2018; Han et al., 2019; Cui et al., 2020). The study by Cui et al. (2020) found that generalized patients with anxiety disorder have increased dALFF in a wide range of brain regions, such as bilateral dorsomedial prefrontal cortex and hippocampus, which is positively related to the severity of symptoms. Another study found that compared to HCs, dALFF was significantly increased in brain regions such as the bilateral thalamus, the bilateral cerebellum posterior lobe, and the vermis in patients with major depressive disorder, and the dALFF value of some brain regions with abnormal dALFF is positively correlated with the severity of major depressive disorder symptoms (Zheng et al., 2021). However, whether there is abnormal dynamic local brain activity in patients with SLE has not been reported.

In the present study, we used ALFF combined with a sliding window approach to calculate dALFF for assessing the temporal variability of intrinsic brain activity in SLE patients with or without CI. We will preprocess fMRI data to calculate sALFF map and dALFF map of each subject and further verify whether the above two indicators are different in SLE patients with or without CI and HCs to explore potential imaging indicators that can be used to identify and evaluate SLE-related CI and provide a new perspective for a more complete understanding of the underlying neuropathological mechanisms of NPSLE. We expected an altered dALFF pattern in patients with SLE compared with HCs. Furthermore, dALFF can detect a subset of potentially abnormal brain activity that is not available with sALFF, which could deepen our understanding of the pathological mechanisms of NPSLE. We also hypothesized that dALFF might be associated with cognitive function.

## Materials and methods

### Participants

A total of seventy-six patients with SLE (thirty-eight with and without CI, respectively) were recruited from the outpatient and inpatient departments of the Rheumatology and Immunology Department of the First Affiliated Hospital of Kunming Medical University.

The inclusion criteria for the case group were: (1) patients diagnosed as SLE according to the 1997 revised American college of rheumatology (ACR) SLE classification criteria; (2) age range from 18 to 50 years; (3) CI was confirmed after Mini-mental State Examination (MMSE) scale assessment (A MMSE score ≤ 26 was identified as CI); (4) right-handedness.

The exclusion criteria for the case group were: (1) patients with connective tissue disorders such as rheumatoid arthritis, systemic sclerosis, primary or secondary Sjögren’s syndrome who meet the ACR classification criteria; (2) patients with epilepsy, severe active mental illness, stroke, traumatic brain injury, history of intracranial surgery, etc. that may interfere with brain structure or functional imaging; (3) patients with a history of drug abuse and alcoholism; (4) women during pregnancy or lactation; (5) patients who have contraindications to MRI (e.g., claustrophobia, metal implants); (6) patients with structural brain abnormalities on conventional T1- or T2-weighted MRI scan.

Thirty-eight gender and age-matched HCs were recruited for this study.

This study has been approved by the Ethics Committee of the First Affiliated Hospital of Kunming Medical University. Before the start of the trial, the subjects and their legal guardians were informed of the trial procedures in detail and they signed the informed consent.

#### Psychological assessment and disease activity index scale

On the day of the MRI examination, a psychiatrist and a rheumatologist, respectively assessed the cognitive function and SLE disease activity index of each SLE patient using the MMSE scale and SLE disease activity index 2,000 (SLEDAI-2k).

### Magnetic resonance imaging data acquisition

An experienced neuroradiologist acquired MRI images of all subjects using a 1.5T MRI scanner with head coils. First, conventional T1WI and T2WI scans were performed to exclude subjects with obvious brain structural abnormalities. No subjects were excluded due to structural brain abnormalities; 3D-MRI uses a 3D-T1-weighted fast phase perturbation gradient echo (3D-T1-fspgr) sequence with the following parameters: repetition time (TR) = 10.5 ms, echo time (TE) = 2.0 ms, inversion time = 350 ms, slice thickness = 1.8 mm and no layer interval, flip angle (FA) = 15°, spatial resolution = 0.94 mm × 0.94 mm × 0.9 mm, scanning matrix = 256 × 256, FOV = 24 cm × 18 cm, layer number = 172, scans cover the entire brain. The resting-state fMRI uses the gradient echo (GRE) sequence of EPI technology, and the specific parameters are as follows: TR = 2,000 ms, TE = 40 ms, NEX = 2.0, imaging matrix = 64 × 64, FOV = 24 cm × 24 cm, FA = 90°, slice thickness = 5 mm, slice interval = 1 mm, slice number = 24, time points = 160, a total of 320 s, and the scanning range covers the whole brain.

The subjects are required to stay awake, rest, lie flat on the examination bed, breathe calmly, fix their head, and minimize the movement of their head and other parts. At the same time, they are required to rest with their eyes closed and try not to do any thinking activities.

### Data preprocessing

BOLD-fMRI data were preprocessed using the DPARSF (Data Processing Assistant for Resting-State fMRI) v4.4 software in the DPABI v6.1 (a toolbox for Data Processing and Analysis for Brain Imaging)^1^ (Yan et al., 2016) software package in the Windows operating system. The specific data preprocessing includes the following: (1) data organization, where the original fMRI data in the format and structure required by DPABI is organized so that the software can automatically recognize and read the fMRI data; (2) input parameters such as time point (160) and TR (2 s); (3) format conversion, where Dicom files are converted to NIfTI files; (4) the first 10 time points are removed. At several time points at the beginning of the scan, due to factors such as unstable gradient magnetic field and patient incompatibility, the image noise is large. To reduce its influence on the overall image, the data of the first 10 time points were removed; (5) slice timing: to correct the errors caused by different acquisition times between each layer; (6) head movement correction: to calculate, the subject’s head movement are reported and corrected. To reduce the impact of head movement on data quality and statistical results, those with translation > 2.0 mm and rotation > 2.0° in the head movement parameters are excluded. (7) The physiological and head movement effects were reduced by removing covariates and linear drift, including white matter and cerebrospinal fluid signal and 24 Friston movement parameters; (8) Register T1 structural image to fMRI: the high resolution of T1 structural image to improve the accuracy of subsequent fMRI data analysis is used; (9) segmentation: the image is segmented into gray matter, white matter, and cerebrospinal fluid; (10) regression of irrelevant covariates and de-linear drift: the influence of machine noise and head movement in the whole brain signal is removed; (11) Filtering: the filtering range of 0.01–0.1 Hz is selected to eliminate the influence of noise other than the BOLD signal frequency; (12) Spatial normalization and resampling: The fMRI images of each subject were registered to the same template for subsequent comparisons. This study used the MNI space-based EPI template and resampled the voxels to 3 × 3 × 3 mm^3^; (13) Smoothing: to improve the signal-to-noise ratio, the FWHM selected in this study is “4 4 4.”

### Static and dynamic amplitude of low-frequency fluctuation computation

The ALFF reflects the low-frequency oscillation strength of spontaneous brain activity (Zang et al., 2007). The calculation of sALFF is to Fourier transform the time series eigenvalues of a certain voxel to frequency space to obtain the average amplitude value in a specific frequency range. The frequency range in this study is 0.01–0.10 Hz. A sliding-window method (Leonardi and Van De Ville, 2015) was used to calculate the dALFF maps by the Dynamic and Stability Analyses module in DPABI v6.1 (Yan et al., 2016) software. Existing research believes that the choice of window length will affect the results of sliding window-related research (Shakil et al., 2016), and window sizes in the range of 40–100 s can capture the dynamic changes of the brain well (Zalesky and Breakspear, 2015). The *f*min is interpreted as the minimum frequency of the time series. To minimize spurious fluctuations, the minimum window length should be higher than 1/*f*min (Li et al., 2018). Thus, we select the optimal window width of 50 TRs (100 s) and a window with a step size of 1 TR (2 s) to dynamically intercept the fMRI time signal and finally apply the variance to quantify the difference between the average ALFF values to obtain the mean dALFF value. The standard deviation (SD) of the measured values in all time windows was calculated by using the mean value of dALFF to quantitatively analyze and compare the time dynamic characteristics of dALFF. Finally, a *z*-transformation was conducted on the individual sALFF maps and dALFF maps to generate normally distributed zsALFF and zdALFF maps.

### Statistical analysis

All demographics and clinical characteristics data were analyzed by SPSS 23.0 software package. Non-parametric K-S test was used to test the normality of the data, and the data distributed normally were expressed as mean ± standard deviation ($\bar{x}$± s), while the data with skewed distribution were expressed as median (p25%, p75%). One-way analysis of variance (ANCOVA) was used to compare the age differences among the three groups. Chi-square test (χ^2^) was used to compare the gender composition ratio among the three groups. Duration of the disease, MMSE score, and drug accumulation were compared between the SLE with CI and SLE without CI groups by Mann–Whitney *U*-test. Independent-sample *t*-test was used to compare SLEDAI-2K scores in SLE with and without CI groups. For each test statistic, a two-tailed probability value of < 0.05 was considered as significant. To further investigate differences in changes in dALFF temporal variability, data statistics module in DPABI software was used to conduct a voxel-based ANCOVA to compare the difference of dALFF value across the three groups with age, gender, and head motion (mean framewise displacement, FD) as covariate, multiple comparisons were performed using the LSD method. To reduce the family-wise error rate (FWER), we adopted a conservative approach with Gaussian random field (GRF) theory (voxel level *P* < 0.01, cluster level *P* < 0.01). *Post-hoc* pairwise comparisons were performed by a two-sample *t*-test if ANCOVA yielded significant results and a GRF correction was used at the cluster threshold of *P* < 0.01 and a voxel-wise threshold of *P* < 0.01. The sALFF of the three groups were compared using the same method. Each brain region was found to be significant different in dALFF and sALFF between SLE with CI group and SLE without CI group or HCs group was identified as the region of interest (ROI). We used DPABI software (Yan et al., 2016) to extract the mean value of dALFF and sALFF of each ROI, respectively. Spearman correlation analysis was applied between dALFF and sALFF for each ROI and MMSE scores in the SLE with CI group by using GraphPad 8.0.2 software.^2^ The threshold for significance was set at *P* < 0.05.

## Results

### Demographics and clinical characteristics

The demographics and clinical characteristics of the three groups are summarized in Table 1. No significant differences were detected among SLE patients with CI, SLE patients without CI, and HCs in age and gender. SLE patients with CI and SLE patients without CI groups showed no significant difference in duration of disease, SLEDAI-2k scores, anti-Sm antibody, anti-U1RNP antibody, drug accumulation of glucocorticoids, hydroxychloroquine, and cyclophosphamide, but significant difference in anti-dsDNA antibody and MMSE scores. For SLE patients with CI, the MMSE scores ranged from 17 to 26.

**TABLE 1:** Results of demographic and clinical data of SLE patients with cognitive impairment group, SLE patients without cognitive impairment, and healthy controls.

	SLE with cognitive impairment (*n* = 38)	SLE without cognitive impairment (*n* = 38)	HCs (*n* = 38)	Statistical	*P*-value
Gender (female/male)	4/34	4/34	4/34	χ^2^ = 0	1.000
Age (year)	30.50 ± 6.45	29.55 ± 6.03	31.89 ± 7.53	*F* = 1.18	0.31
Duration of disease (month)	12.00 (3.00, 24.00)	12.50 (1.66, 32.75)	NA	*U* = 707.50	0.88
SLEDAI-2k	10.42 ± 5.07	9.58 ± 6.26	NA	*t* = 0.65	0.52
MMSE scores	24.00 (21.00, 25.00)	29.50 (29.00, 30.00)	NA	*U* <0.01	<0.01
**Antibody, *n* (%)**					
Anti-Sm antibody	20 (52.63)	21 (55.26)	NA	χ^2^ = 0.05	0.82
Anti-dsDNA antibody	28 (73.68)	19 (50.00)	NA	χ^2^ = 4.52	0.03
Anti-U1RNP antibody	12 (31.58)	14 (36.84)	NA	χ^2^ = 0.23	0.63
Anti-P0 antibody	23 (60.53)	16 (42.11)	NA	χ^2^ = 2.58	0.11
GC accumulation (g)	2.55 (0, 10.61)	1.64 (0.26, 12.09)	NA	*U* = 657.50	0.50
HCQ accumulation (g)	0 (0, 3.30)	0.35 (0, 11.70)	NA	*U* = 604.00	0.18
CTX accumulation (g)	0 (0, 1.00)	0 (0, 0.25)	NA	*U* = 628.50	0.25

SLEDAI-2k, Systemic Lupus Erythematosus disease activity index 2000; MMSE, Mini-mental State Examination; NA, not applicable; GC, glucocorticoid; HCQ, hydroxychloroquine; CTX, cyclophosphamide.

### Static amplitude of low-frequency fluctuation result

**FIGURE 1:**
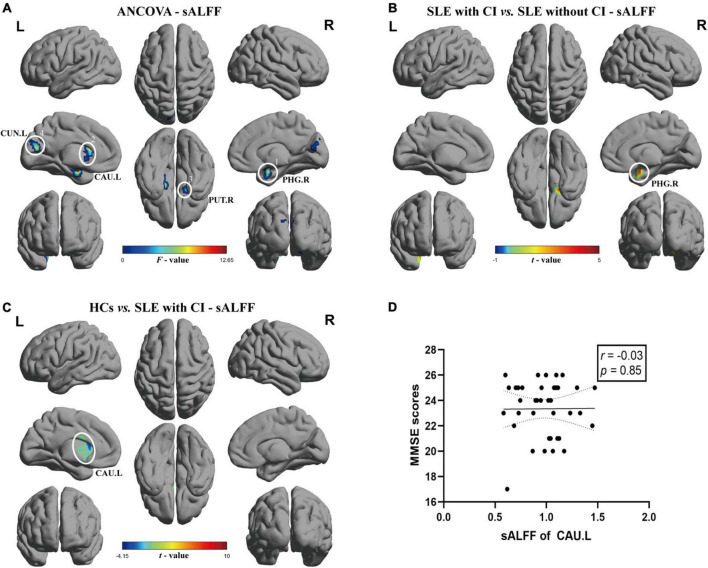
Brain regions with significant differences of sALFF among SLE with CI, SLE without CI and HCs. Specifically, **(A)** ANCOVA results of sALFF among three groups (voxel *P* < 0.01, cluster *P* < 0.05, controlling for age, gender and head motion, GRF corrected), **(B,C)** Differences between the groups were calculated using *post-hoc* analysis based on a two-sample *t*-test (voxel *P* < 0.01, cluster *P* < 0.05, controlling for age, gender, and head motion, GRF corrected). **(D)** Correlation analysis showed no significant correlation was found between sALFF and MMSE scores in SLE with CI groups. Color bars represent statistical value; the solid lines and dashed lines represented the linear regression fitted line and 95% confidence interval of the Spearman correlation analysis, respectively, SLE with CI, systemic lupus erythematosus patients with cognitive impairment; SLE without CI, systemic lupus erythematosus patients without cognitive impairment; HCs, healthy controls; sALFF, static amplitudes of low-frequency fluctuation; MMSE, Mini-mental State Examination; L, left hemisphere; R, right hemisphere; PHG, parahippocampal gurys; CAU, caudate nucleus; PUT, lenticular nucleus, putamen; CUN, Cuneus.

The results of ANCOVA revealed that subjects in the SLE with CI, SLE without CI, and HC groups exhibited significant sALFF variability in the right parahippocampal gyrus, left caudate nucleus, right putamen and left cuneus (voxel *P* < 0.01, cluster *P* < 0.05, controlling for age, gender and head motion, GRF corrected) (Table 2 and Figure 1A). Compared to the SLE without CI, the right parahippocampal gyrus exhibited higher sALFF in the SLE with CI group (Figure 1B). Compared to the HC, the left caudate nucleus exhibited increased sALFF in the SLE with CI group (Figure 1C). There was no significant difference in the sALFF between the HC group and the SLE without CI group.

**TABLE 2:** Regions changes in sALFF in SLE with CI group compared with the SLE without CI and HCs groups.

Brain regions	Cluster size (voxels)	xyz-peak (MNI)	*F*/*t*-value
**ANCOVA**			
Right parahippocampal gyrus	207	–18/–3/–21	12.65
Left caudate nucleus	135	–12/12/6	11.78
Right putamen	59	27/12/-6	7.85
Left cuneus	71	0/-75/18	8.10
**HCs vs. SLE with CI**			
Left caudate nucleus	169	–12/12/6	–4.151
**SLE with CI vs. SLE without CI**			
Right parahippocampal gyrus	137	15/6-24	3.80

Statistical significance was set at voxel P < 0.01, cluster P < 0.05, controlling for age, gender and head motion, GRF corrected.

ALFF, amplitudes of low-frequency fluctuation; SLE with CI, Systemic lupus erythematosus patients with cognitive impairment; SLE without CI, Systemic lupus erythematosus patients without cognitive impairment; HCs, Healthy controls; MNI, Montreal Neurological Institute.

### Dynamic amplitude of low-frequency fluctuation variance result

**FIGURE 2:**
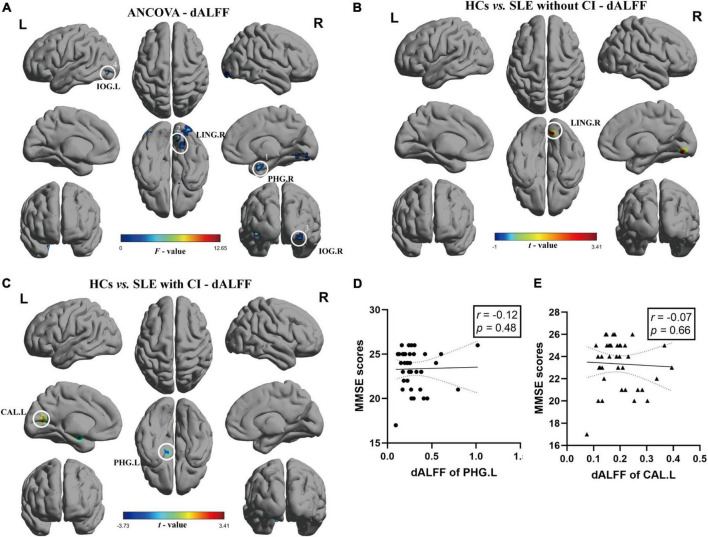
Brain regions with significant differences of dALFF among SLE with CI, SLE without CI, and HCs. Specifically, **(A)** ANCOVA results of dALFF among three groups (voxel *P* < 0.01, cluster *P* < 0.05, controlling for age, gender, and head motion, GRF corrected). **(B,C)** Differences between the groups were calculated using *post-hoc* analysis based on a two-sample *t*-test (voxel *P* < 0.01, cluster *P* < 0.05, controlling for age, gender, and head motion, GRF corrected). **(D,E)** Correlation analysis showed no significant correlation was found between dALFF and MMSE scores in SLE with CI groups. Color bars represent statistical value; the solid lines and dashed lines represented the linear regression fitted line and 95% confidence interval of the Spearman correlation analysis, respectively, SLE with CI, systemic lupus erythematosus patients with cognitive impairment; SLE without CI, systemic lupus erythematosus patients without cognitive impairment; HCs, healthy controls; dALFF, dynamic amplitudes of low-frequency fluctuation; MMSE, Mini-mental State Examination; L, left hemisphere; R, right hemisphere; PHG, para hippocampal gurys; LING, lingual gyrus; IOG, inferior occipital, putamen; CAL, calcarine fissure and surrounding cortex.

The results of ANCOVA revealed that participants in the SLE with CI, SLE without CI, and HCs groups exhibited significant dALFF variability in the right parahippocampal gyrus, right lingual gyrus, and bilateral inferior occipital gyrus (voxel *P* < 0.01, cluster *P* < 0.05, controlling for age, gender, and head motion, GRF corrected) (Table 3 and Figure 2A). Compared to the HCs, the right lingual gyrus exhibited reduced dALFF in the SLE without CI group (Figure 2B). Compared to the HC, the right parahippocampal gyrus exhibited increased dALFF and left calcarine fissure, and the surrounding cortex exhibited reduced dALFF in the SLE with CI group (Figure 2C). No significant differences were found in the dALFF between the SLE with CI group and the SLE without CI group.

**TABLE 3:** Regions changes in dALFF in SLE with CI group compared with the SLE without CI and HCs groups.

Brain regions	Cluster size (voxels)	xyz-peak (MNI)	*F*/*t*-value
**ANCOVA**			
Right parahippocampal gyrus	29	18/3/–30	10.87
Right lingual gyrus	60	12/-72/-12	13.60
Right inferior occipital gyrus	37	33/–90/–15	7.44
Left inferior occipital gyrus	34	–36/–78/–6	13.67
**HCs vs. SLE with CI**			
Right parahippocampal gyrus	59	–9/–6/–18	–3.73
Left calcarine fissure and surrounding cortex	48	–15/–69/6	3.41
**HCs vs. SLE without CI**			
Right lingual gyrus	56	12/–72/–12	4.12

Statistical significance was set at voxel P < 0.01, cluster P < 0.05, controlling for age, gender, and head motion, GRF corrected.

ALFF, amplitudes of low-frequency fluctuation; SLE with CI, Systemic lupus erythematosus patients with cognitive impairment; SLE without CI, Systemic lupus erythematosus patients without cognitive impairment; HCs, Healthy controls; MNI, Montreal Neurological Institute.

### Correlation analysis results

There was no significant correlation between sALFF, dALFF values and MMSE scores for each ROI in the SLE with CI group (Figures 1D, 2D,E).

## Discussion

So far, this is the first fMRI study in patients with SLE that uses both the sALFF and dALFF analysis. We also performed correlation analyses on the sALFF and dALFF abnormalities and MMSE scores. Our study found that compared with HCs and SLE patients without CI, SLE patients with CI showed different characteristics of brain intrinsic functional connectivity strength and stability, among which, the sALFF of SLE with CI was increased in the right parahippocampal gyrus compared with SLE patients without CI, and increased in the left caudate nucleus compared with HCs. In terms of temporal variability in the amplitude of local brain activity, the dALFF of SLE with CI was increased in the right parahippocampal gyrus, but reduced in the left calcarine fissure and surrounding cortex compared with HCs. Compared to the HCs, the right lingual gyrus exhibited reduced dALFF in the SLE without CI group. We did not find a correlation between abnormal sALFF and dALFF in SLE patients with CI and their cognitive performance.

Several previous studies have investigated the intensity of intrinsic brain activity in patients with SLE within the scope of sALFF. These studies found that non-NPSLE patients showed a decrease of sALFF in the bilateral precuneus and an increase in the right cuneus and the right calcarine fissure surrounding cortex, respectively (Yu H. et al., 2019). Another study found increased standardized ALFF in the left inferior temporal gyrus and left putamen in non-NPSLE patients compared with HCs (Yu Y. et al., 2019). A previous study by our research group found that, compared with HCs group, the ALFF values of the bilateral postcentral gyrus in the non-NPSLE group were lower than those in the HCs group, while the ALFF values in the bilateral inferior temporal gyrus, left putamen, and bilateral precuneus were higher than those in the HCs group (Piao et al., 2021). Our study included SLE patients with CI and found that these patients had two brain regions with different sALFF values than HCs and SLE patients without CI. The caudate nucleus participates in cognitive processes by stimulating the correct movement patterns and selecting appropriate secondary targets based on the assessment of the action outcome (Grahn et al., 2008). The parahippocampal gyrus is located in the medial under the occipital and temporal lobes, is the main cortical input to the hippocampus, is considered to play an important role in high cognitive functions including memory coding retrieval and visuospatial processing, and is an important center for memory processing (Lin et al., 2021). This study found that the sALFF in the above two brain regions of SLE patients with CI was higher than that of HCs and SLE patients without CI, respectively, suggesting that the local brain activity intensity of these two brain regions increased, which may be related to the compensation of cognitive dysfunction. Similar results have also been reported in studies of major depressive disorder (Liu et al., 2014), and amnestic CI (Zhang et al., 2021) and its specific mechanism still needs further research.

Previous studies assumed that ALFF was static throughout the rs-fMRI scan, but recent studies confirmed that ALFF is time-varying (Fu et al., 2018). To better understand the mechanism of cognitive dysfunction in patients with SLE, we used a sliding window approach for the first time on the basis of sALFF to study the brain dynamics describing temporal changes in energy expenditure (temporal variability of dALFF). We found that SLE patients with CI did indeed have temporal ALFF alterations. Specifically, compared with HCs, the SLE patient group with CI had a brain region that showed increased (right parahippocampal gyrus) and decreased (left calcarine fissure and surrounding cortex) dALFF, respectively. Brain dynamics reflect functional capabilities of the nervous system (Kucyi et al., 2017), and may more sensitively reflect disruption of cognitive function in multiple diseases (Cui et al., 2020; Lu et al., 2020; Zheng et al., 2021). The results obtained by this innovative dALFF method have led us to realize that in addition to the abnormal local activity intensity of the brain, there are also changes in the stability of local activity dynamics in patients with SLE. As mentioned above, the right parahippocampal gyrus is involved in memory and other cognitive functions (Lin et al., 2021), and the abnormal enhancement of the intensity variability of brain functional activity in this brain region may be one of the mechanisms of CI in patients with SLE. Similar results have been reported in chronic obstructive pulmonary disease patients with semantic-memory impairments. Calcarine fissure and the surrounding cortex is responsible for receiving and transmitting visual signals (Huang et al., 2020), changes in the stability of functional activity in this brain region have been reported in studies on non-NPSLE (Chen et al., 2021), but its exact effect on CI needs more studies to clarify. Notably, the brain regions with abnormal sALFF and dALFF values did not overlap, which may reflect that the intensity and stability of brain activity affect cognitive function in patients with SLE through different neuropathological pathways, suggesting that the assessment of dALFF can complement the results of traditional sALFF and promote our understanding of the pathological mechanism. Future studies can explore the possibility of dALFF as a new biomarker for SLE patients with CI by imaging machine learning or receiver operator characteristic (ROC) curve analysis.

Unfortunately, we did not find a correlation between abnormal sALFF values, dALFF values, and cognitive function scores in the SLE patient group with CI, other than possibly due to our relatively small sample size and the use of only one scale to assess cognitive function in patients with SLE, it may also be related to the properties of fMRI and the pathogenesis of SLE-related CI. Researchers have tried to use neuroimaging, immunology (Varley et al., 2020), bioinformatics (Geng et al., 2020), and other methods to study NPSLE. Considering the diversity of its clinical manifestations, the pathogenesis of NPSLE is generally considered to be the result of the interaction of multiple pathological processes. Some studies have found that certain autoantibodies and cytokines may lead to cerebrovascular lesions and/or interfere with neuronal connections by mediating immune responses (Bertsias and Boumpas, 2010; Borowoy et al., 2012; Schwartz et al., 2019), and genetic factors and disruption of the blood–brain barrier may also be involved in the pathogenesis of NPSLE (Schwartz et al., 2019). The confounding caused by this heterogeneity in etiology may cause differences in the performance of individual brain functions, and ultimately the sALFF and dALFF values of the regions with the most obvious differences between groups are not significantly related to individual cognitive function. On the other hand, the changes of sALFF and dALFF may be more sensitive than MMSE in distinguishing SLE from CI, that is, the changes of these two indicators can help to identify subclinical CI patients in SLE earlier. In the future, we also need to group the etiology as much as possible through the detection of autoantibodies (such as antiphospholipid antibodies, etc.) and cerebrospinal fluid and obtain more representative subgroups to study the correlation between CI and brain function in a more targeted manner. Future research can perform other scales that reflect neuronal function such as Montreal Cognitive Assessment (MoCA), mini-Cog, etc. and analyze the correlation with sALFF and dALFF values, which may reveal more about the relationship between cognitive function and its fMRI indicators in patients with SLE. In addition to ALFF, fALFF, ReHo, and other fMRI indicators can also complement ALFF to help us understand NPSLE more comprehensively.

This study also has some limitations. First of all, the field strength of the magnetic resonance scanner used in this study (1.5T) is lower than that currently mainstream in brain imaging research (3.0T), so our results may be biased due to the smaller signal-to-noise ratio. We actually started this research 10 years ago and continued the same 1.5T MRI scanner and scanning parameters to build the database. In this study, we conducted strict quality control, including manual visual inspection and software quality control. We also hope to use new magnetic resonance scanners in the future to obtain more accurate results. Secondly, we only used a window length and step size recommended by previous studies and did not use other methods to validate the main results. Finally, this study only applied dALFF to examine the temporal dynamics of local brain activity. However, future work can also explore the changes in fMRI indicators such as dfALFF and dReHo in patients with SLE.

## Conclusion

In conclusion, SLE patients with CI not only have abnormal brain activity intensity but also have changes in brain activity stability. By describing the dynamic changes in the intrinsic brain activity, it provides a new idea for elucidating the pathophysiological mechanism of cognitive dysfunction in SLE. However, more research and validation with multimodal data are needed to determine the link between the severity of CI and brain activity in patients with SLE.

## Data availability statement

The raw data supporting the conclusions of this article will be made available by the authors, without undue reservation.

## Ethics statement

The studies involving human participants were reviewed and approved by the Institutional Review Board of Kunming Medical University. The patients/participants provided their written informed consent to participate in this study. Written informed consent was obtained from the individual(s) for the publication of any potentially identifiable images or data included in this article.

## Author contributions

YY, RZ, and FZ were responsible for the management of the research and writing the article. RB and SLi were responsible for recruiting and following up with the patients. RC and SLu were responsible for the consultation of the research. JX was responsible for the whole research and article. All authors contributed to the article and approved the submitted version.

## Footnotes

1
http://rfmri.org/dpabi

2
https://www.graphpad.com/
